# The effectiveness of Pictorial Representation of Illness and Self Measure (PRISM) for the assessment of the suffering and quality of interpersonal relationships of patients with chronic pain

**DOI:** 10.1186/s13030-021-00223-0

**Published:** 2021-11-20

**Authors:** Mitsunao Tomioka, Masako Hosoi, Tomona Okuzawa, Kozo Anno, Rie Iwaki, Hiroshi Kawata, Chiharu Kubo, Nobuyuki Sudo

**Affiliations:** 1 Department of Psychosomatic Medicine, Graduate School of Medical Sciences, Kyushu University, 3-1-1 Maidashi, Higashi-ku, Fukuoka City, Fukuoka, 812-8582 Japan; 2 Department of Psychosomatic Medicine, Kyushu University Hospital, 3-1-1 Maidashi, Higashi-ku, Fukuoka City, Fukuoka, 812-8582 Japan; 3 Multidisciplinary Pain Center, Kyushu University Hospital, 3-1-1 Maidashi, Higashi-ku, Fukuoka City, Fukuoka, 812-8582 Japan; 4 Center for Dementia Related-Diseases, Konan Medical Center, 1-5-16 Kamokogahara, Higashinada-ku, Kobe City, Hyogo 658-0064 Japan; 5 Department of Psychosomatic Medicine, Kyushu Central Hospital of the Mutual Aid Association of Public School Teachers, 3-23-1 Shiobaru, Minami-ku, Fukuoka City, Fukuoka, 815-8588 Japan; 6 Nakamura Gakuen University, 5-7-1 Befu, Jounan-ku, Fukuoka City, Fukuoka, 814-0198 Japan

**Keywords:** Pictorial representation of illness and self measure, Chronic pain, Patient/physician relationship, Patient/significant others relationship

## Abstract

**Background:**

Pictorial Representation of Illness and Self Measure (PRISM) is a tool that can be used to visualize and evaluate the burden of suffering caused by an illness. The aim of this study was to identify which aspects of the burden of chronic pain patients are associated with Self/illness separation (SIS), an indicator of the magnitude of suffering. We also examined the effectiveness of PRISM for evaluating changes in the relationships between patients and their medical care and significant others due to our inpatient treatment.

**Methods:**

Seventy-two patients with chronic pain who were outpatients or admitted to the Department of Psychosomatic Medicine completed PRISM, depression and anxiety scales, and three types of pain-related self-assessment questionnaires (Brief Pain Inventory, Short-form McGill Pain Questionnaire, and Pain Catastrophizing Scale). Outpatients were queried at the time of outpatient visits and inpatients at the time of admission. In addition to PRISM disks related to illness, we asked each patient to place disks related to things important to them and their medical care. Of the inpatients, 31 did PRISM at the time of discharge. Among the reported important factors, which significant other was placed at the time of admission and discharge was evaluated. The distances of self/medical care separation (SMcS) and self/significant others separation (SSoS) were measured.

**Results:**

Of the 21 scales measured, 10 showed a significant correlation with SIS. Factor analysis of these 10 scales extracted three factors, Life interferences, Negative affects, and Pain intensity. The SMcS and SSoS distances were shorter at discharge than at admission.

**Conclusions:**

PRISM for patients with chronic pain is an integrated evaluation method that reflects three aspects of pain. By adding medical care and significant others to the usual method of placing only illness on the sheet it became possible to assess changes in the quality of interpersonal relationships.

## Introduction

Chronic pain is a disease that causes great damage to a patient’s mental and physical health. The prevalence of chronic pain has been reported to be 22.5 to 40% of all adults [1–4]. It is important to evaluate the clinical outcomes of hospital clinical practice promptly and accurately.

The pain experience is a complex product of various experiences. Not only the pain intensity, but also the negative emotional experience caused by it, the catastrophic cognition of pain, and the disabilities due to the pain that are experienced at the same time. Therefore, multidimensional evaluation of the effect of treatment for patients with chronic pain is recommended. The Initiative on Methods, Measurement, and Pain Assessment in Clinical Trials (IMMPACT) [5] listed six core areas to consider when conducting clinical trials: (1) pain; (2) physical functioning; (3) emotional functioning; (4) participant rating of improvement and satisfaction with treatment; (5) symptoms and adverse events; and (6) participant disposition. In addition, IMMPACT II [6] presented a standard evaluation method for each domain.

In routine clinical practice, it can be difficult for patients suffering from pain to fill out long questionnaires. Depending on the patient’s readiness, it may be difficult to assess the psychological aspects of their disease. An example is the orthopedic field. It deals with common physical illnesses such as low back pain and knee pain, but such patients are often reluctant to have psychological evaluations. Therefore, a simple evaluation method that can be tolerated by and provide a clear picture of the patient is required. The visual analogue scale and face scale of pain are well known, simple pain evaluation methods, but they do not include scales for the deterioration of quality of life (QOL) or psychological function and are not multidimensional, but evaluate only one aspect of pain.

**Fig. 1:**
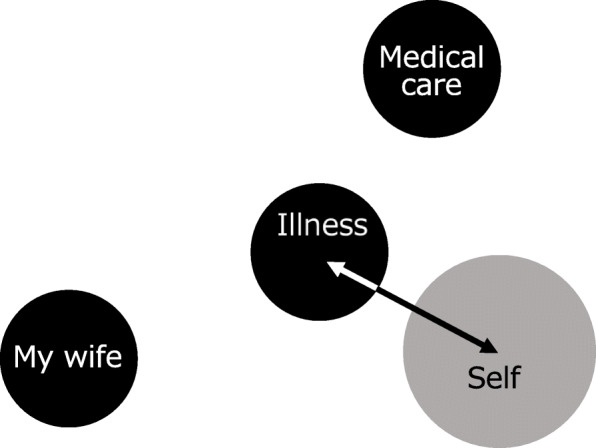
An example of a PRISM task. The colored disks have been replaced with grayscale. The length of the arrow indicates the Self/Illness Separation (SIS). PRISM = Pictorial Representation of Illness and Self Measure

Furthermore, pain is a subjective experience and the patient’s suffering is noteworthy. The Pictorial Representation of Illness and Self Measure (PRISM) has been reported from the viewpoint of evaluating the suffering of patients with chronic diseases [7, 8], and its usefulness has been reported for the evaluation of diseases associated with pain [9]. In PRISM, a circle representing oneself is drawn on A4 size paper (Fig. 1). The patient is asked to place a disk representing the illness on the paper. The distance between the illness and oneself (Self/Illness Separation: SIS) can be used to evaluate the magnitude of the impact of the illness on the patient [8]. In many of the studies to date, PRISM has been used as a tool to assess the suffering caused by illness [10, 11]. PRISM is a simple measure based on a visual metaphor. Although there is no ‘gold standard’ measure of suffering, there is substantial evidence, including quantitative and qualitative data summarized in a systematic review [11], that PRISM measures suffering due to illness. In other words, the closer the illness disc is to the self, the more difficult it is for the patient to control their symptoms, and the more their illness erodes their lives. In a study of pain patients, the greater the severity of the illness and the intensity of the pain, the shorter the SIS, which means that one’s self and the illness are placed closer together. In addition, Büchi et al. [7] reported a method (PRISM+) of placing disks representing people, things, and affairs that are important to the patient in addition to the one representing the illness. It has been reported that this method helps the therapist and patient better understand the patient’s experience of illness and living conditions and that it helps build the patient-therapist relationship [7]. Kassardjian et al. [9] placed significant others, such as family members and partners, in addition to illness, and showed that shorter distances between them and one’s self had a more positive impact on the patient. We aim to improve the quality of our patient-therapist relationships through supportive and empathic psychotherapy for patients with pain. We hope that such treatment will have a positive effect on the quality of the interpersonal relationships of our patients that will lead to an improvement in their QOL. Establishing good relationships with the medical staff is related to the success of subsequent step-by-step psychosomatic medical treatments such as transactional analysis and mindfulness-based therapies.

Based on these considerations, the current study investigated the possibility that PRISM can be used as an integrative measure for patients with chronic pain. We hypothesized that various pain outcomes, including pain intensity, physical functioning, and emotional functioning, would contribute the SIS of the PRISM in patients with chronic pain.

PRISM can also reflect the quality of relationships with others [9]. We measured patients’ appraisals of their medical care and of significant others. We hypothesized that these relationships would be improved by successful supportive inpatient treatment.

The first aim of this study is to clarify which factors contribute to the SIS of PRISM in patients with chronic pain. The second aim is to examine if PRISM enables longitudinal assessment of the patient when inpatient treatment is given.

## Methods

### Participants

The participants were recruited from inpatients and outpatients treated for chronic pain in the Department of Psychosomatic Medicine, Kyushu University Hospital. Eligibility criteria included: 1) a three month or more history of pain; 2) an ability to read and write Japanese; 3) being 20 years old or older; and 4) a willingness to participate in the study. Exclusion criteria included: 1) the presence of psychotic symptoms; 2) an inability to read due to visual impairment; and 3) lack of consent for study participation. All patients, both inpatient and outpatient, who met the criteria were invited to participate, and all accepted.

Data collection was done between November 2005 and January 2011. Outpatients were asked to complete the pain related measures and did PRISM while waiting for their consultation. Inpatients completed the measures and PRISM within 10 days after admission, then again within 10 days before discharge. This study was done with the approval of the Kyushu University Institutional Review Board for Clinical Research.

### Measures

#### The PRISM task

Participants were shown a white A4-size paper with a yellow disk 7 cm in diameter at the bottom right-hand corner (Fig. 1). Each participant was asked to imagine that the paper represented his/her life at that moment and the yellow disk represented the participant’s “self”. They were given a red paper disk, 5 cm in diameter, and asked to imagine that it represented their “illness”. The instructions used were exactly those in previous validation studies [12]. They were then asked, “Where would you put your illness—the red disk—in your life at this moment?” (note: the red disk represented the patient’s illness rather than pain specifically). The evaluator then asked the patient to place a person, thing, or affair that was important to them. The methodology of this study enables them to choose their preferred color for each disc (5 cm in diameter) freely from 12 colors (PRISM-Kyudai Version: PRISM-KV).

Finally, the evaluator instructed the participants to think about how they feel about their current medical care, select one of the colored disks, and place it on the sheet. After completing the PRISM task, patients were asked why they had placed the medical care and significant other disks where they were. The distance between the “self” and “illness” disks (ie SIS) was measured from the center of the “self” disk to the center of the “illness” disk. Similarly, the distance between the disk representing their self and the significant other (Self/Significant others Separation: SSoS) and the distance between those representing their self and their medical care (Self/Medical care Separation: SMcS) was measured. The possible range was 0 to 27 cm. Participants did this task on admission and discharge. The PRISM evaluator identified the highest valued person and the order of priority, as follows: (1) most important person at present, (2) current spouse or partner, (3) mother, father, or both, (4) children, (5) siblings, (6) family (including multiple members).

#### Depression and anxiety

The level of depression was evaluated by the Center for Epidemiologic Studies Depression scale (CES-D) [13, 14]. This scale consists of twenty items and has a range of 0 to sixty. Anxiety level was evaluated by use of the State Trait Anxiety Inventory (STAI) [15]. This scale has two aspects of anxiety: state anxiety and trait anxiety. Both scales consist of twenty items, each with a score range of 20 to 80. The Japanese version of STAI has been validated [16].

#### Japanese version of the brief pain inventory (BPI)

A Japanese version of the Brief Pain Inventory [17] was used to assess pain intensity and pain interference. Participants were asked to rate each item on a scale of 0–10. Pain intensity was assessed by four items (worst pain, least pain, average pain, and current pain,). Pain interference was assessed on seven domains of functioning, including mood, walking, work, and relation with others. The original BPI covers pain intensity and interference in the last twenty-four hours, while another study has expanded the term to one week [18]. We adopted the later method for this study in order allow the assessment of usual or characteristic pain and to avoid unreliability in our measurement due to possible daily fluctuations in pain. Our former study displayed good internal consistency for the intensity and interference subscales (Cronbach’s alpha = 0.84 and 0.89, respectively) [19].

#### Japanese version of the short-form McGill pain questionnaire (SF-MPQ)

The SF-MPQ is used to assess the sensory and affective dimensions of pain experience [20]. It consists of fifteen items (eleven sensory and four affective) and includes a 6-point present pain index (PPI) and a visual analogue scale (VAS), which measure overall pain intensity. The reliability and validity of the Japanese version of the SF-MPQ have been reported [21].

#### Japanese version of the pain catastrophizing scale (PCS)

The PCS consists of thirteen items referring to thoughts and feelings that a person may have when experiencing pain [22]. Participants were asked to indicate the degree to which they are experiencing pain on a 5-point Likert scale. Pain-related catastrophizing has been defined as “an exaggerated negative orientation toward pain stimuli and pain experience”. This scale assesses three catastrophizing dimensions (Rumination, Magnification and Helplessness). Like the original version, the Japanese version has good internal consistency (Cronbach’s alpha > 0.80; Magnification’s alpha = 0.65) [23].

#### Inpatient treatment

**Fig. 2:**
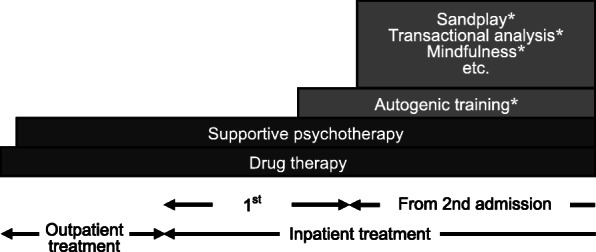
Stepwise psychosomatic therapy: from outpatient to inpatient treatment. *Administered according to the patient’s condition

We provide general medications, such as Non-Steroidal Anti-Inflammatory Drugs (NSAIDs), anticonvulsants, antidepressants, anxiolytics, and hypnotics, to treat the pain of patients with chronic pain from the time of outpatient treatment (Fig. 2). When a patient is admitted for inpatient treatment, the supportive psychotherapy that was done previously is administered more intensively, and stepwise psychosomatic therapy is provided. The aim of our initial inpatient treatment is to determine the daily status of the clinical symptoms of the patient without active medical care. In the first stage of inpatient treatment, a life review is done to assess the patient’s condition, including the patient’s pain status and environmental background. A study examining factors that aggravate the pain of patients with chronic pain has pointed to problems with their relationships with parents in early childhood [24]. In our inpatient treatment we do our interviews with a supportive and empathic attitude and endeavor to build a highly reliable patient-therapist relationship. Autogenic training was done for patients (*N* = 18, 40.9%) who were judged to have established a relationship of trust. The point of discharge is when we can better understand the psychopathology of the patient.

### Data analysis

Statistical analysis was done with SPSS ver.14.0 J for Windows (SPSS Japan Inc., Tokyo, Japan). We first computed Pearson’s correlation coefficients between SIS and the pain related scales. Factor analysis was done to identify the latent factors present in the variables that were significant in the bivariate correlation. At the same time, factor scores were computed through the factor analysis. Pearson’s correlation coefficients were computed for the SIS and factor scores in order to specify factors correlated with SIS. Changes in PRISM-KV variables (SIS, SMcS, and SSOS) from admission to discharge were analyzed by Wilcoxon signed rank test. The frequency of positive and negative SIS differences between discharge and admission was tested by chi-square test. The relation between ΔSMcS and ΔSSoS (difference between admission and discharge) was analyzed by calculating the Spearman’s rank correlation coefficient.

## Results

**Fig. 3:**
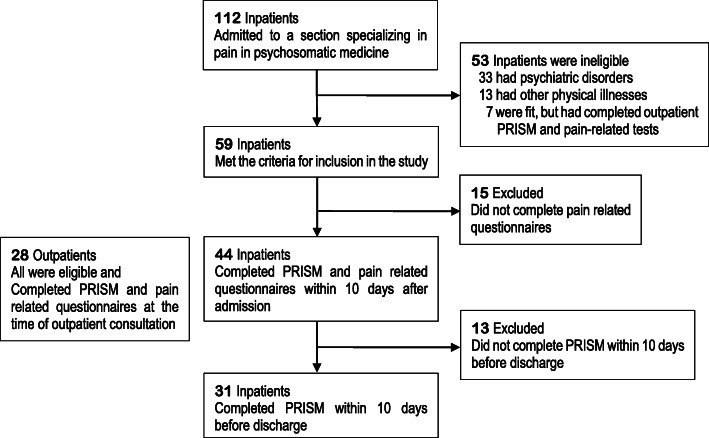
Participant flowchart

One hundred and twelve patients were admitted for treatment in our section specializing in pain within the Department of Psychosomatic Medicine, of whom 59 met the inclusion criteria. Forty-four of them (74.6%) completed PRISM-KV and the other pain-related questionnaires. Of the 44, 31 (70.5%) completed PRISM-KV at both admission and discharge. Of the outpatients, 28 who met the criteria were asked to cooperate in the research, and valid data were obtained from all 28 (Fig. 3).

Demographic data of the 72 studied patients is shown in Table 1: 54 (75%) were female, the mean age was 48.6 years (SD = 11.7), 45.8% were unable to work due to pain, the mean duration of chronic pain was 92.1 months (range 7–456 months), and the most common sites of pain were the shoulder (69.4%), leg/foot (63.9), neck (61.1%), upper back (61.1%), and lower back (61.1%).

**Table 1:** Demographic data for the 72 patients studied

Variable	Number or Mean
Sex (male/female)	18 / 54
Mean age (years)	48.6 (SD = 11.7)
Employment	
Full-time work	5
Part-time work	5
Household	18
Retired	1
Unable to work due to pain	33
Unable to work not due to pain	10
Duration of chronic pain (months)	92.1 (min = 7, max = 456)
VAS of pain (mm)	71.3 (SD = 21.4)
Location of chronic pain (multiple replies)	
Head / face	36 (50.0%)
Neck	44 (61.1%)
Shoulder	50 (69.4%)
Arm / hand	38 (52.8%)
Chest	24 (33.3%)
Abdomen	24 (33.3%)
Upper back	44 (61.1%)
Low back	44 (61.1%)
Buttocks	23 (31.9%)
Leg / foot	46 (63.9%)
Others	13 (18.1%)
CES-D	26.4 (SD = 12.6)
STAI-State	53.2 (SD = 11.6)
STAI-Trait	53.8 (SD = 12.2)

Note. VAS = Visual Analogue Scale, CES-D = Center for Epidemiologic Studies Depression Scale, STAI = State Trait Anxiety Inventory

Computation of the Pearson’s correlation coefficients between SIS and the pain related scales (21 subscales) identified 10 subscales that had a significant relation to SIS (Table 2). Factor analysis of these variables was done to clarify which accounted for the SIS (Table 3). Three factors with eigenvalues of more than 1.00 were extracted by the principal component method with direct oblimin rotation.

**Table 2:** Correlations between SIS and pain related variables

Pain related variables	Correlation coefficients
Negative feelings	
CES-D	−.363**
STAI State	−.389**
STAI Trait	−.404**
Brief Pain Inventory (BPI)	
Worst pain	−.434**
Least pain	−.137
Average pain	−.216
Current pain	−.330**
Interfered with general activity	−.357**
mood	−.308**
walking	−.263*
work	−.310**
relations with others	−.241*
sleep	−.184
enjoyment of life	−.111
Short-form McGill Pain Questionnaire (SF-MPQ)	
Sensory pain	−.126
Affective pain	−.137
Visual Analogue Scale	−.221
Present pain intensity	−.228
Pain Catastrophizing Scale (PCS)	
Rumination	−.187
Helplessness	−.039
Magnification	−.101

Note. SIS = Self Illness Separation, CES-D = Center for Epidemiologic Studies Depression Scale, STAI = State Trait Anxiety Inventory

*P < .05

**P < .01

**Table 3:**
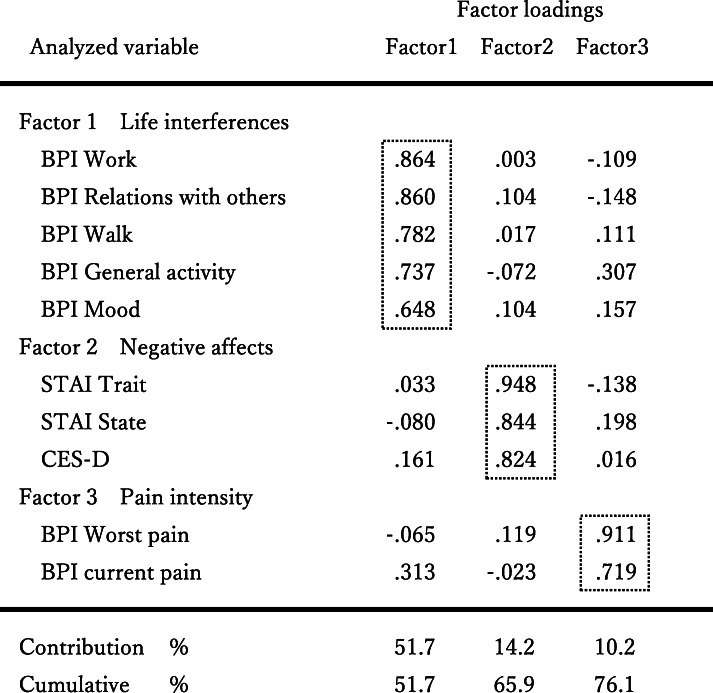
Factor analysis of pain related variables correlated to SIS

Note. Each factor was extracted by the principal component method with direct oblimin rotation. Only the factor pattern matrix is displayed

SIS = Self Illness Separation, BPI = Brief Pain Inventory, STAI = State Trait Anxiety Inventory, CES-D = Center for Epidemiologic Studies Depression Scale

The first factor that had high factor loadings included the work-related interference items BPI and interference with relations with others. We designated this factor “Life interference”. The second factor that had high factor loading included the trait anxiety and state anxiety subscales of STAI and CES-D, respectively. We designated this factor “Negative affects”. The third factor that had high factor loading was the worst pain and current pain items of BPI. We designated this factor “Pain intensity”.

Pearson’s correlation coefficients were calculated for the SIS and factor scores computed in the process of factor analysis (Table 4). The correlation coefficients were − .326 (*P* < .01) for Life interferences and SIS, −.420 (*P* < .01) for negative affects and SIS, and − .392 (*P* < .01) for Pain intensity and SIS.

**Table 4:** Correlation between SIS and the three significant factors extracted in the factor analysis

Factors	Correlation coefficients
Factor 1 Life interferences	−.326**
Factor 2 Negative affects	−.420**
Factor 3 Pain intensity	−.392**

Note. Each factor score was calculated by the factor analysis shown in Table 3. Correlation coefficients were calculated by the factor scores and SIS distances

SIS = Self/Illness Separation

**P < .01

The PRISM evaluator identified eleven spouses, seven parent(s), seven family members, three children, one sibling, and one boss as significant others. Only one patient did not place the same significant other in both PRISMs. Comparison of the PRISM-KV variables at admission and discharge showed statistically significant changes in SMcS and SSoS (Table 5). Both were placed closer to the self-representing circle at discharge than at admission. Table 6 shows the comments on SMcS and SSoS at admission and discharge. The comments of the two participants with the largest positive and negative values are displayed. Participants whose SMcS was shorter at discharge (Δ < 0) than at admission changed from anxious and unclear about their medical care to satisfied with the care. They had a more positive impression of their medical care at discharge.

**Table 5:** Analysis of changes in PRISM variables correlated with inpatient treatment (*N* = 31)

	admission ^a)^ 25–75 ^b)^	discharge ^a)^ 25–75 ^b)^	Z	*P* ^c)^
SIS (cm)	3.50 .40–7.20	3.25 .80–7.00	−1.20	.232
SMcS (cm)	8.30 4.40–12.60	6.00 3.50–10.60	−2.54	.011
SSoS (cm)	7.80 5.90–13.80	6.20 4.60–11.70	−3.08	.002

^a)^ Median, ^b)^ 25th and 75th percentiles, ^c)^ Wilcoxon’s signed rank test

SIS = Self/Illness Separation, SMcS = Self/Medical care Separation, SSoS = Self/Significant others Separation

**Table 6:**
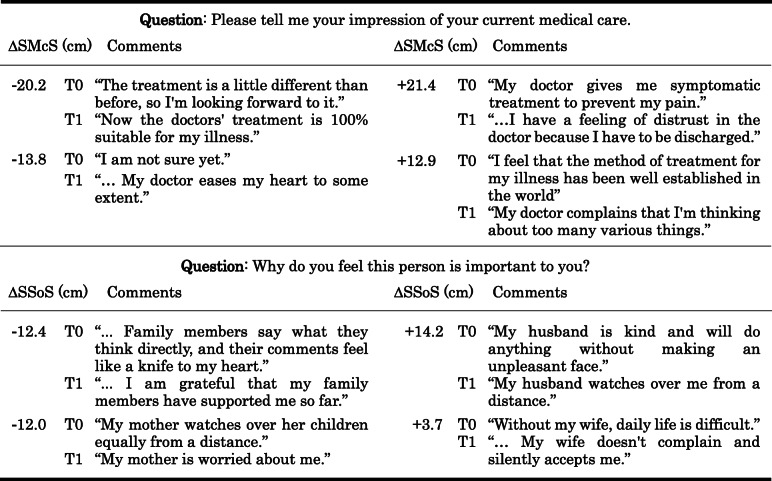
Changes in distance and patient comments on their medical care and significant others discs

A negative Δ value indicates that the medical care or significant others disc is closer to the self at the time of discharge than at the time of admission

*SMcS* Self/Medical care Separation, *SSoS* Self/Significant others Separation, *T0* At admission, *T1* At discharge, *Δ* T1 minus T0

The same was true for SSoS, with it becoming shorter at discharge than at admission, when the importance of significant others was reported by the participant to be greater. There were no significant changes in SIS.

The difference between the SIS values at admission and discharge was calculated (discharge minus admission) and encoded as positive (*n* = 17) or negative (*n* = 11). Zero (*n* = 2) was invalidated and excluded. When the encoded data was analyzed with Chi square testing, the frequency bias was not significant (χ2(1) = 1.29, *p* = 0.26).

No significant correlation of variables at admission and discharge was found between ΔSMcS and ΔSSoS (Spearman r = − 0.147, *p* = 0.440). Furthermore, the amount of change was encoded as positive or negative to investigate if the SSoS of the participants whose SMcS was shortened was also shortened. Two participants with zero change were excluded. SMcS was shortened for 24 of the 28 participants, of whom 19 had shortened SSoS.

## Discussion

This study is the first to report that PRISM, an indicator of suffering, can be used to assess three psychosocial factors associated with chronic pain. These three elements of IMMPACT [5] have been identified in clinical practice as important outcomes in chronic pain management. We found that SIS is defined by these three factors, showing its utility as a simple visual evaluation method for the quick assessment of patient suffering by medical staff engaged in the treatment of chronic pain, e.g. physical therapists, occupational therapists, orthopedic surgeons, physicians, nurses, as well as psychologists and psychiatrists. Furthermore, we have shown for the first time that SMcS and SSoS are useful as methods for evaluating the interpersonal relationships of patients with chronic pain.

### SIS of PRISM as an integrative tool

The SIS of patients with chronic pain in this study showed a significant correlation with 10 of the 21 pain-related subscales. Factor analysis of these scales extracted three factors: “Life interference”, “Negative affects”, and “Pain intensity”. The correlation coefficients for the SIS and the factor scores of these factors were significant.

The above pain-related aspects are considered to be elements corresponding to pain, physical functioning, and emotional functioning, which are reported to be important factors in IMMPACT [5]. Further investigation is needed to clarify if or how the other three domains (participant rating of improvement and satisfaction with treatment; symptoms and adverse events; and participant disposition) are related to the PRISM variables.

This study yielded results consistent with previous PRISM validation studies [11, 12]. In them, the most consistent and significant negative correlation of SIS is reported to be depression and pain. Unexpectedly, in some cases, depression and health-related quality of life did not correlate with SIS. Suffering due to illness is thought to be influenced by numerous variables; however, none of the variables measure suffering directly, thus the correlations with suffering can be expected to be significant, but modest [11, 12]. Medical staff can intuitively understand the status of their patients with chronic pain when they see the PRISM image. Therefore, we believe that it is a useful, integrative, visualized measure that reflects many factors at the same time.

A study by Kassardjian et al. [9] examining the association between PRISM and psychosocial factors associated with the medical condition of patients with chronic pain showed a significant relation between SIS and pain sensation, physical function, and mental state. The results of the present study are consistent with their results. Although, their study showed a significant negative relation between pain catastrophizing and SIS, the current study did not. In the current study, the PRISM task measured ‘my illness’, whereas Kassardjian et al. measured ‘my pain’. This methodological difference may be responsible for the different results. It is possible that our patients are highly alexithymic: they may be aware of the distress from pain, but may not be aware of their catastrophizing. It is also possible that their pain behaviors may be being used to attract the attention of others or to avoid aversive interpersonal relations, which increases their catastrophizing. Due to these confounding factors, the two variables may not have a linear relation.

### SMcS and SSoS as indicators of interpersonal relationships

In response to our supportive and empathetic psychotherapy, the position of medical care and significant others on PRISM-KV changed from admission to discharge. Both discs were closer to the discs representing the participants themselves (Self) after treatment than before treatment. Kassardjian et al. [9] showed that better relationships with patients exist when the partner and family on PRISM are placed closer to themselves. Prior to this study, no attempts have been made to put feelings about medical care on PRISM. Of the four comments on SMcS, all clearly expressed a change in their impression of their doctors, which indicates to us that the SMcS reflects their interpersonal relationship with medical care. The participants of this study showed that their relationship with medical staff members changed for the better and that they considered the staff members more reliable. It was also shown that inpatient treatment improved the relationship with significant others. Considering that our inpatient treatment was centered on supportive psychotherapy, it is not surprising that the patient/therapist relationship was reflected in the distance between medical care and self on PRISM-KV. The shorter distances reflected a better relationship between the patient and the therapist, and there is a possibility that experiencing intimacy with someone in a hospital influences the relationship between the patient and their significant others. Our data do not show a correlation between ΔSMcS and ΔSSoS, so the involvement of other confounding factors, i.e. interpersonal relationships with other patients, must be examined in future studies.

We previously reported that patients with chronic pain admitted to the Department of Psychosomatic Medicine had perceived lower care, higher overprotection, and affectionless control-type care from their parents at an early age than did a painless control group [24]. It has also been suggested that relationships with significant others are strongly associated with pathology [25]. Patients treated with a patient-oriented coping skill approach with spousal support have been reported to benefit more from treatment than did patients treated with a patient-only coping skill approach [26]. Our patients were asked “Why do you feel this person is important to you?” after placing the disc of significant others. We feel that our results show that our patients place great importance to their interpersonal relationships. Improvement of relationships with significant others by patients with chronic pain is an important treatment step. This is the first study to show that this modified PRISM can be used to evaluate such changes in interpersonal relationships.

This study did not show significant change in the correlation of SIS with inpatient treatment. Previous longitudinal studies of PRISM report a sensitive response of SIS to treatment [12, 27–29]. In contrast, other reports did not reflect the effectiveness of treatment [30, 31]. Gielissen et al. [30] reported that the position of a cancer disc did not change despite successful treatment of fatigue in cancer patients. Töndury et al. [31] showed that patients with chronic urticaria showed no change in SIS despite improved symptoms and QOL.

The tertiary medical facility inpatient management of the patients of this study focused on psychosomatic assessment and the establishment of a therapeutic relationship, which may be the reason there was little change in pain suffering. To our knowledge, no longitudinal study of SIS in chronic pain has yet been done. Further investigation is needed to determine if successful psychosomatic interventions can change the SIS in chronic pain patients.

### Limitations

This study has the following limitations. First, there were only 72 participants, and only for 31 of them were we able to examine the effectiveness of treatment. There may be some selection bias because not all subjects could be included in the analysis. To generalize the results, it will be necessary to compare the change from before to after treatment for all consecutive participants. Second, a more detailed understanding of the properties of medical care and significant others when taken up as PRISM variables is needed. The selection of significant others was made by the PRISM evaluator, but it would be better if the patients were to do it themselves. Strategic examinations are needed on what factors are involved in the change of the distance of SSoS on PRISM, such as support for treatment and a change of partner. Third, we only applied qualitative analysis to determine the relationship with the medical care staff or significant others. Further investigation using a questionnaire on the relationship directly with various members of the medical care staff or significant others is needed to clarify that the distance actually reflects interpersonal relationships.

Despite these limitations, we found our evaluation method based on PRISM to be useful in the evaluation of the suffering of patients with chronic pain. Our investigation sheds light on the possibility that this modified PRISM can be used to assess the improvement of patient-therapist and patient-significant other relationships in response to supportive and empathic psychotherapy.

## Conclusion

PRISM for patients with chronic pain is an integrated evaluation method that reflects three aspects of pain: “Life interference”, “Negative effects”, and “Pain intensity”. By placing medical care and significant others discs in addition to the usual method of placing an illness disc on the sheet, it was possible to assess change in the quality of interpersonal relationships, which is an important factor in the pathology of patients with chronic pain.

## Data Availability

The datasets used and analyzed during the current study are available from the corresponding author on reasonable request.

## Abbreviations

PRISM: Pictorial Representation of Illness and Self Measure
PRISM-KV: PRISM-Kyudai Version
IMMPACT: Initiative on Methods, Measurement, and Pain Assessment in Clinical Trials
QOL: Quality of life
SIS: Self/Illness Separation
SSoS: Self/Significant others Separation
SMcS: Self/Medical care Separation
CES-D: Center for Epidemiologic Studies Depression scale
STAI: State Trait Anxiety Inventory
BPI: Brief Pain Inventory
SF-MPQ: Short-Form McGill Pain Questionnaire
PCS: Pain Catastrophizing Scale
NSAIDs: Non-Steroidal Anti-Inflammatory Drugs
