# Correction to: The effectiveness of Pictorial Representation of Illness and Self Measure (PRISM) for the assessment of the suffering and quality of interpersonal relationships of patients with chronic pain

**DOI:** 10.1186/s13030-021-00230-1

**Published:** 2022-01-25

**Authors:** Mitsunao Tomioka, Masako Hosoi, Tomona Okuzawa, Kozo Anno, Rie Iwaki, Hiroshi Kawata, Chiharu Kubo, Nobuyuki Sudo

**Affiliations:** 1 Department of Psychosomatic Medicine, Graduate School of Medical Sciences, Kyushu University, 3–1-1 Maidashi, Higashi-ku, Fukuoka, 812–8582 Japan; 2 Department of Psychosomatic Medicine, Kyushu University Hospital, 3–1-1 Maidashi, Higashi-ku, Fukuoka, 812–8582 Japan; 3 Multidisciplinary Pain Center, Kyushu University Hospital, 3–1-1 Maidashi, Higashi-ku, Fukuoka, 812–8582 Japan; 4 Center for Dementia Related-Diseases, Konan Medical Center, 1–5-16 Kamokogahara, Higashinada-ku, Kobe, Hyogo 658–0064 Japan; 5 Department of Psychosomatic Medicine, Kyushu Central Hospital of the Mutual Aid Association of Public School Teachers, 3–23-1 Shiobaru, Minami-ku, Fukuoka, 815–8588 Japan; 6 Nakamura Gakuen University, 5–7-1 Befu, Jounan-ku, Fukuoka, 814–0198 Japan

**Correction to: BioPsychoSocial Med 15, 22 (2021).**

**https://doi.org/10.1186/s13030-021-00223-0**

Following publication of the original article [1], the authors identified an error in Fig. 3 and Table 6.

**Fig. 3:**
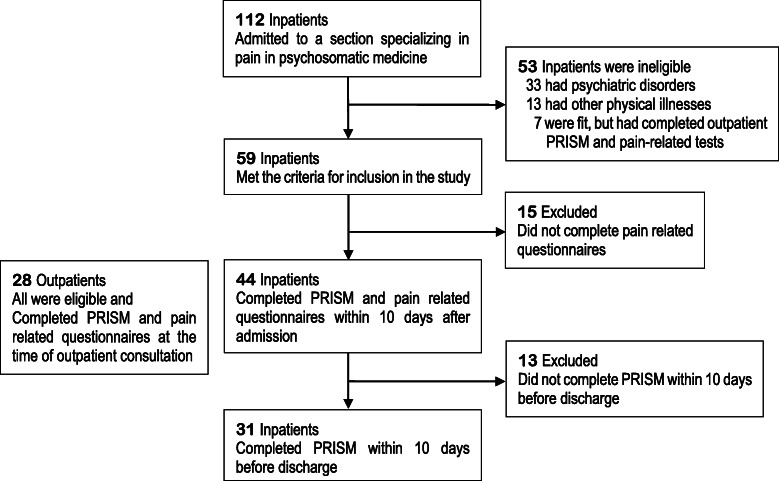
Participant flowchart

**Table 6:**
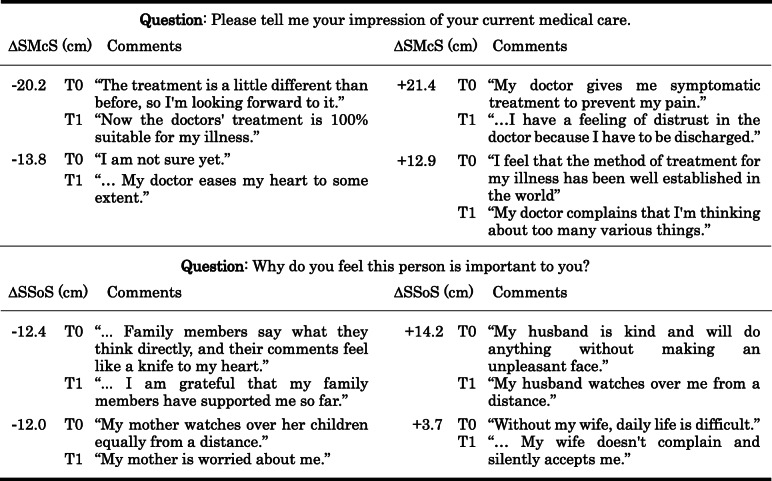
Changes in distance and patient comments on their medical care and significant others discs

A negative Δ value indicates that the medical care or significant others disc is closer to the self at the time of discharge than at the time of admission

SMcS = Self/Medical care Separation. SSoS = Self/Significant others Separation. T0: At admission. T1: At discharge. Δ = T1 minus T0

In the old Fig. 3, there was a layout problem in the upper right part of the figure. The word “tests” in the sentence “7 were fit. But it had completed outpatient PRISM and pain-related tests” was out of the box.

In the old Table 6, eight respondents were shown for the two questions, each person’s comment consisted of two comment parts before (T0) and after (T1). The old table cannot distinguish between them. The author put the replaced shapes in the table in the extended metafile so that it wouldn’t get out of shape.

The correct figure and table have been included in this correction, and the original article has been corrected.
